# Perinatal Choline Supplementation Promotes Resilience Against Progression of Alzheimer's Disease‐Like Brain Transcriptomic Signatures in *App* ^NL‐G‐F^ Mice

**DOI:** 10.1111/acel.70148

**Published:** 2025-10-13

**Authors:** Thomas A. Bellio, Andre Krunic, Mary S. Campion, Rohan Dupaguntla, Adam Labadorf, Thor D. Stein, Honghuang Lin, Tiffany J. Mellott, Jan K. Blusztajn

**Affiliations:** ^1^ Department of Pathology and Laboratory Medicine Boston University Chobanian and Avedisian School of Medicine Boston Massachusetts USA; ^2^ Department of Pharmacology, Physiology, and Biophysics Boston University Chobanian and Avedisian School of Medicine Boston Massachusetts USA; ^3^ Department of Neurology Boston University Chobanian and Avedisian School of Medicine Boston Massachusetts USA; ^4^ Bioinformatics Program Boston University Boston Massachusetts USA; ^5^ VA Boston Healthcare System Boston Massachusetts USA; ^6^ Department of Veterans Affairs Medical Center Bedford Massachusetts USA; ^7^ Boston University Alzheimer's Disease Research Center Boston University Chobanian and Avedisian School of Medicine Boston Massachusetts USA; ^8^ Department of Medicine University of Massachusetts Chan Medical School Worcester Massachusetts USA

**Keywords:** Alzheimer disease, choline, computational biology, resilience

## Abstract

Alzheimer's disease (AD)—the leading cause of dementia—has no cure, inadequate treatment options, and a limited understanding of prevention measures. We have previously shown that perinatal dietary supplementation with the nutrient choline ameliorates cognitive deficits and reduces amyloidosis across the brain in *App* ^NL‐G‐F^ AD model mice. Here, we analyzed transcriptomic abnormalities in these mice and tested the hypothesis that they may be attenuated by perinatal choline supplementation (PCS). Wild‐type (WT) and *App* ^NL‐G‐F^ dams consumed a diet containing 1.1 (control) or 5 g/kg (supplemented) of choline chloride from 2 weeks prior to mating until weaning. At 3, 6, 9, or 12 months of age, the offspring RNA was sequenced in the hippocampus and cerebral cortex. As compared to WT, the *App* ^NL‐G‐F^ mice reared on the control diet had age‐dependent upregulation of expression of mRNAs and lncRNAs related to inflammation and reduced expression of mRNAs related to neuronal function. As compared to *App* ^NL‐G‐F^ mice on the control diet, PCS *App* ^NL‐G‐F^ mice increased expression of synaptic genes and downregulated inflammation‐related genes starting at 6 months in the cortex; increased expression of GABAergic function and ATP metabolism genes, and decreased expression of inflammatory genes in the hippocampus at 12 months. These changes counteracted the effects of *App* ^NL‐G‐F^ genotype seen in mice on the control diet. The expression of many of these choline‐protected genes correlated with clinical dementia rating, inflammation, and tauopathy in human postmortem dorsolateral prefrontal cortex AD samples, indicating their relevance to the disease process. The results suggest that adequate choline intake could be a preventive strategy for AD.

## Graphical abstract

Choline‐protected genes correlated with clinical dementia rating, inflammation, and tauopathy in human AD samples, suggesting that adequate choline intake could be a preventive strategy for Alzheimer's disease (AD).

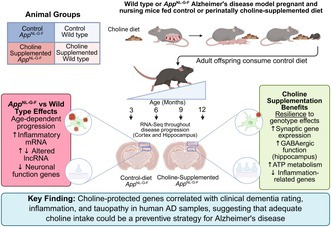

## 1 Introduction

Alzheimer's disease (AD)—the most common form of age‐associated dementia worldwide—is characterized by behavioral and cognitive deficits clinically, and neuropathologically by the accumulation of extracellular amyloid‐beta (Aβ) plaques, intraneuronal neurofibrillary tangles (NFTs), neuroinflammation, and synapse loss. Despite years of research, there is no cure, and treatment options remain limited, underscoring the need for preventative approaches for this illness. Indeed, while AD risk increases with age—11% prevalence in individuals over the age of 65 overall, reaching 33% in people over the age of 85—the disease is not an inevitable consequence of old age as a significant proportion of population exhibits resistance to the AD‐related neuropathologic changes or resilience to them by not developing clinical symptoms despite neuropathology (2025 Alzheimer's Disease Facts and Figures 2025; Fang et al. 2025; Latimer et al. 2024) indicating that genetic, environmental, or lifestyle factors, including nutrition, may reduce this risk. One of the best‐characterized nutritional approaches supporting normal brain function is the adequate supply of the essential nutrient, choline (Blusztajn et al. 2017), a metabolic precursor of (1) phosphatidylcholine, the most abundant lipid in mammalian cell membranes (van der Veen et al. 2017), (2) acetylcholine, a neurotransmitter controlling muscle contraction, sympathetic nervous system activity, brain development, attention, and learning, and (3) *S*‐adenosylmethionine, the source of methyl groups for the enzymatic methylations of DNA and histones, thus modulating the patterns of gene expression (Zeisel and da Costa 2009). High intake of choline during the perinatal period is neuroprotective later in life in multiple animal models of neuronal dysfunction, including those related to aging, epilepsy, prenatal alcohol exposure, as well as genetic diseases such as Rett and Down syndromes. Moreover, perinatal or lifelong choline supplementation in AD mouse models significantly ameliorates multiple AD‐related phenotypes (Bellio et al. 2024; Chartampila et al. 2024; Mellott et al. 2017; Velazquez et al. 2019). Despite the importance of choline in health and disease, 75%–90% of the population does not reach their daily reference intake value for this nutrient, indicating an unmet nutritional need (Wallace et al. 2018).

We have recently reported that perinatal choline supplementation in the *App* ^NL‐G‐F^ AD mouse model prevented spatial‐ and fearful learning and memory deficits and reduced brain amyloid Aβ42 deposition in the amygdala, cortex, and hippocampus (Bellio et al. 2024). Here, we explored the effects of the *App* ^NL‐G‐F^ genotype and perinatal choline supplementation at 3‐, 6‐, 9‐, and 12‐months of age on gene expression in the hippocampus and cortex. The *App* ^NL‐G‐F^ model uses a humanized version of the Aβ portion of *App* and incorporates the Swedish (KM670/671NL), Arctic (E693G), and Beyreuther/Iberian (I716F) mutations within the *App* gene (Saito et al. 2014). Together, the humanization and mutations of *App* lead to amyloid plaque accumulation and neuroinflammation starting around 2 months of age. These neuropathologies are associated with age‐ and cell type‐dependent abnormalities in gene expression patterns and permitted the discovery of the so‐called “plaque‐induced genes” (PIGs) (W.‐T. Chen et al. 2020). The pathological and behavioral phenotypes in these mice are modifiable by not only our perinatal choline supplementation but also by voluntary exercise (S. Li et al. 2025) and pharmacological interventions (Pauls et al. 2021). In this study, we found that PCS corrects the abnormal expression of genes within several core AD‐related pathways, including synaptic dysfunction and inflammation, throughout disease progression, thus providing its possible molecular mechanisms of action. In an accompanying paper, we provide evidence that PCS modulates gene expression by altered patterns of DNA methylation, suggesting that the latter mechanism is mediated by choline's action as a methyl group donor. Moreover, we found that many of the choline‐protected genes identified in our mouse model are associated with the clinical progression of AD in humans, suggesting that choline targets core mechanisms underlying disease severity.

## 2 Results

### 2.1 *App* ^NL‐G‐F^ Mice Show Upregulated Inflammation and Decreased Synaptic Function Dependent on Time and Brain Region

**FIGURE 1:**
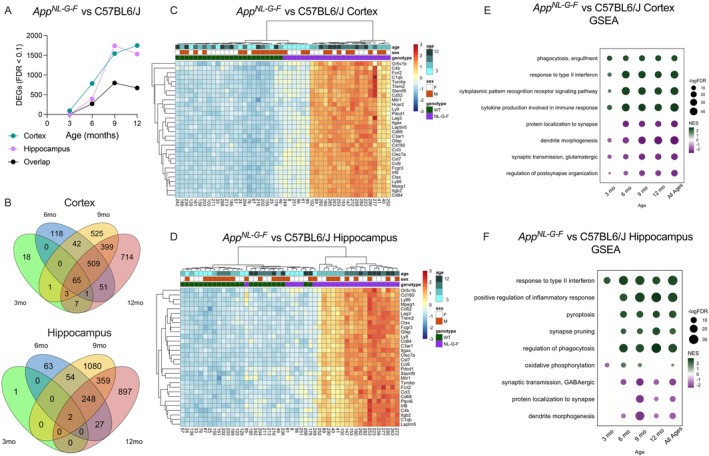
*App* ^NL‐G‐F^ mice show progressive inflammation and synaptic dysfunction in the cortex and hippocampus. (A) Total number of differentially expressed genes (DEGs) between control diet WT and *App* ^NL‐G‐F^ mice over time in the cortex and hippocampus (FDR < 0.1, *n* = 6 per age). (B) Overlap between DEGs in the hippocampus and cortex is shown. Overlap of DEGs in the cortex and hippocampus over time reveals a strong overlap between DEGs starting at 6 months. Hierarchical clustering of the top 30 DEGs by FDR in the (C) cortex and (D) hippocampus reveals distinct transcriptomic differences between WT and *App* ^NL‐G‐F^ mice. (E) Pathway enrichment in the cortex reveals progressive inflammation and synaptic dysfunction as early as 3 months. (F) Pathway enrichment in the hippocampus reveals progressive inflammation and synaptic dysfunction starting at 6 months.

We first set out to identify the transcriptomic differences between wild‐type (WT) and *App* ^NL‐G‐F^ mice. We found an increased number of differentially expressed genes (DEGs) between wildtype and *App* ^NL‐G‐F^ mice with age in the cortex and hippocampus when controlling for sex (Figure 1A) reaching over 1500 (approximately 7% of all detected genes) by the age of 9 and 12 months in each of the brain regions (Supporting Information S1). Notably in the hippocampus, the number of DEGs at 3 months was lower compared to the cortex, before sharply increasing at later time points. This is in line with the progression of amyloid pathology in *App* ^NL‐G‐F^ mice, which typically begins in the neocortex (Bellio et al. 2024). We next compared the overlap between DEGs in different age groups within the same brain region (Figure 1B). In the cortex, 65 genes were shared at all time points, including *Gfap, Cd68*, and *C1qa* (Figure S2A,C,E). The largest number of unique DEGs was found at 12 months, which included glutamatergic receptors such as *Gria2* and *Grm5* (Figure S2G–H). In the hippocampus, the number of DEGs began to rise at 6 months, and DEGs overlapping between 6 and 12 months included many of the same core inflammatory genes in the cortex (Figure S2B,D,F). Interestingly, there were many DEGs specific to 9 and 12 months in the hippocampus. At 9 months, these DEGs included glutamatergic receptors *Gria4* and *Grin2a*, and typically showed a more rapid decline by 9 months in App^NL‐G‐F^ mice, before WT mice reached similar levels by 12 months, indicating acceleration of normal age‐related changes in App^NL‐G‐F^ mice (Figure S2I,J). Genes only differentially expressed at 12 months typically remain stable with aging in WT mice, but show steady age‐dependent decline in AD mice. One notable gene following this pattern was *Cntn4*, a hippocampal synaptic plasticity gene implicated in AD (Figure S2K; Oguro‐Ando et al. 2021). Taken together, this suggests that the decline in synaptic gene expression in App^NL‐G‐F^ mice involves both acceleration of normal aging and unique AD‐specific changes. When stratifying the analyses by sex, we did not observe significant differences between males and females in general (Figures 1C,D and 3B,C), with genes of the core inflammatory pathways remaining consistently upregulated in App^NL‐G‐F^ mice compared to WT in both sexes (Figure S2L). Hierarchical clustering of the DEGs in the cortex and hippocampus showed a clear separation of samples by genotype when using the top 30 DEGs (Figure 1C,D). Moreover, samples clustered by age, with younger *App* ^NL‐G‐F^ mice closer in expression to WT, and a gradient of expression as animals age. Likewise, gene‐set enrichment revealed a strong age‐dependent upregulation of pathways involved in microglial signaling, cytokine production, and complement activation in both brain regions (Figure 1E,F). Additionally, the expression of genes within pathways representing synaptic signaling and function was decreased over time, correlating with increased inflammation. These pathways were dysregulated at an earlier age in the cortex compared to the hippocampus. To validate our transcriptomic findings, we performed western blotting to quantify protein levels of reactive astrocyte marker GFAP and pan‐microglial marker IBA1 by western blot. In both tissues, there was a marked increase in expression of these proteins, consistent with mRNA levels (Figure S3).

**FIGURE 2:**
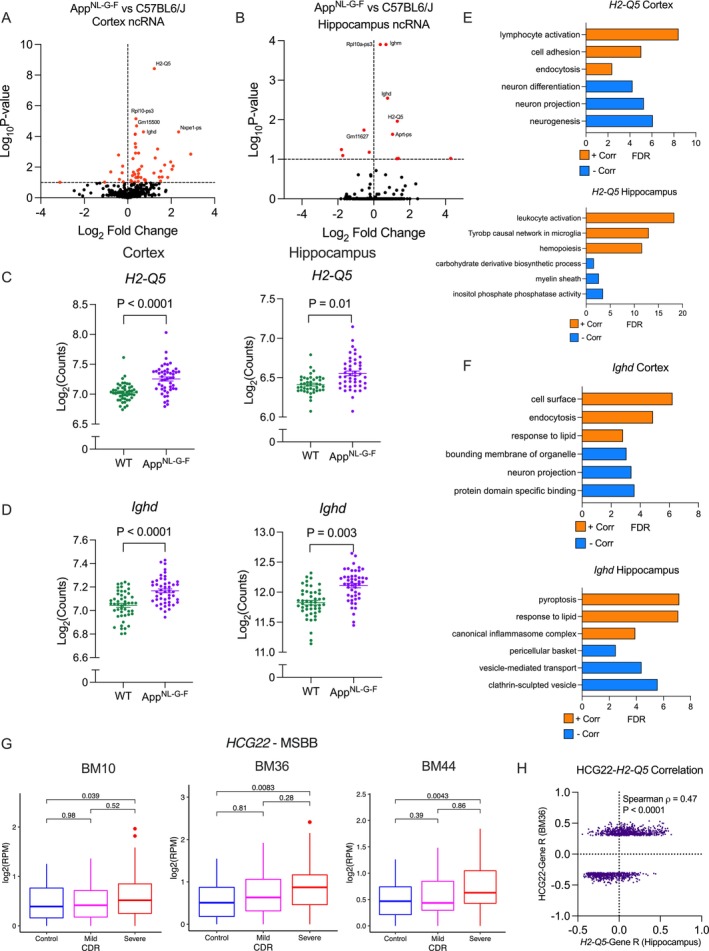
*App* ^NL‐G‐F^ mice have altered lncRNA expression. Volcano plot demonstrating changes in lncRNA expression in (A) cortex and (B) hippocampus of lncRNA transcripts. (C) Expression of *H2‐Q5* is upregulated in *App* ^NL‐G‐F^ mice in the cortex and hippocampus (*n* = 48). (D) Expression of *Ighd* is upregulated in *App* ^NL‐G‐F^ mice in the cortex and hippocampus (*n* = 48). (E) Pathway enrichment of the top 100 mRNAs positively (orange) and negatively (blue) correlated with *H2‐Q5* in the cortex and hippocampus. (F) Pathway enrichment of the top 100 mRNAs positively (orange) and negatively (blue) correlated with *Ighd* in the cortex and hippocampus. (G) *HCG22*, a lncRNA located within the human HLA locus, is upregulated with increasing severity of dementia in BM10, BM36, and BM44. (H) Correlation of lncRNA–mRNA R values between human *HCG22* and murine *H2‐Q5* suggests similar cellular function.

In addition to coding RNA, we probed changes in long non‐coding RNA (lncRNA) due to *App* ^NL‐G‐F^ genotype (Lauretti et al. 2021). Controlling for age and sex, we found 53 differentially expressed lncRNA species in the cortex (Figure 2A), and 13 differentially expressed lncRNAs in the hippocampus (Figure 2B). Two of the top lncRNAs upregulated in both the cortex and hippocampus were *H2‐Q5* (Cortex FDR = 3.79 × 10^−9^, Hippocampus FDR = 0.01, Figure 2C) and *Ighd* (Cortex FDR = 5.06 × 10^−5^, Hippocampus FDR = 0.003, Figure 2D). To identify putative functions of these lncRNAs, we performed a Spearman correlation between the individual lncRNAs and all mRNA species. We then performed pathway enrichment on the top 100 most positively and negatively correlated mRNAs. *H2‐Q5* expression was positively associated with lymphocyte activation in both brain regions (Figure 2E). *Ighd* was positively associated with genes enriched for lipid metabolism, pyroptosis, and inflammasome function, and was negatively correlated with clathrin‐coated vesicle function (Figure 2F). While lncRNAs are often not conserved between species, similar functional orthologs may exist in other species. To answer this question, we consulted an atlas of lncRNA changes in the Mount Sinai Brain Bank (MSBB) to identify lncRNA changes in the human equivalent HLA and Igh loci (C. Chen et al. 2025). Interestingly, we identified *HCG22*, a lncRNA located within the human HLA locus, that was upregulated with increasing severity of dementia in Brodmann areas (BM) 10, 36, and 44 (Figure 2G). As the strongest effect size in humans was in the parahippocampal gyrus (BM36), we compared lncRNA–mRNA correlation coefficients between *HCG22* and *H2‐Q5* to see if they were associated with similar pathways. There was a significant positive correlation between lncRNA–mRNA *R* values in humans and mice, suggesting *HCG22* and *H2‐Q5* fill similar functional roles (Spearman rho = 0.47, *p* < 0.0001, Figure 2H).

### 2.2 Perinatal Choline Supplementation Alters the Trajectory of Gene Expression During AD‐like Pathology Progression

**FIGURE 3:**
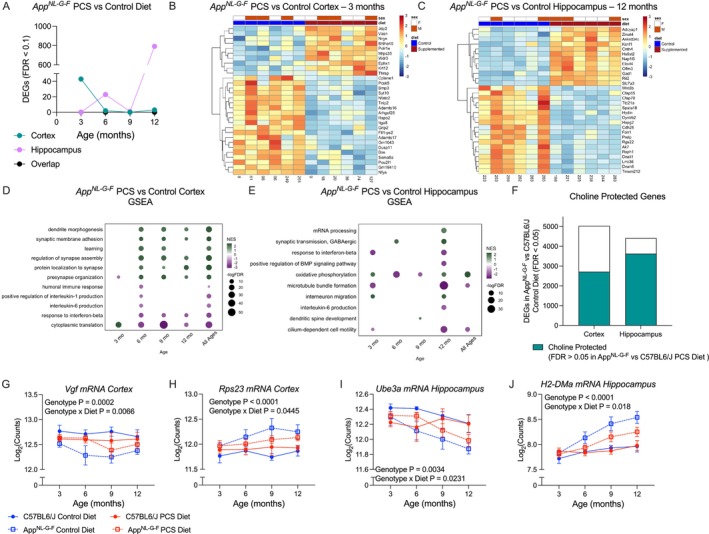
Perinatal choline supplementation can alter gene trajectory over AD progression. (A) Total number of differentially expressed genes (DEGs) between *App* ^NL‐G‐F^ mice fed a PCS or control diet over time in the cortex and hippocampus (FDR < 0.1, *n* = 6). (B) Hierarchical clustering of the top 30 DEGs at 3 months in the cortex reveals transcriptomic differences between PCS and control diet *App* ^NL‐G‐F^ mice. (C) Hierarchical clustering of the top 30 DEGs at 12 months in the hippocampus reveals a distinct effect of diet. (D) Pathway enrichment reveals upregulated synaptic function and downregulated inflammation and oxidative phosphorylation over disease progression in the cortex. (E) Pathway enrichment reveals alterations in oxidative phosphorylation, as well as increased synaptic function and decreased inflammation at 12 months in the hippocampus. (F) Intersection of DEGs (FDR < 0.05, *n* = 24) in control diet *App* ^NL‐G‐F^ mice versus WT and genes not differentially expressed (FDR > 0.05, *n* = 24) controlling for age in PCS *App* ^NL‐G‐F^ mice versus WT. (G–J) Expression of choline responsive genes in the over disease progression reveals blunting of the effect of *App* ^NL‐G‐F^ genotype (*n* = 6). Data shown as mean ± SEM.

We hypothesized that PCS may alter the transcriptome of *App* ^NL‐G‐F^ mice in the cortex and hippocampus across disease progression. Using an FDR cutoff of 0.1, we found the highest number of PCS‐responsive DEGs in the cortex at 3 months (Figure 3A,B, Supporting Information S2). In contrast, the hippocampus had a sizable number of DEGs at 12 months, with 788 meeting the FDR cutoff (Figure 3A,C). In WT mice fed a PCS diet, few DEGs were observed, indicating a strong genotype‐by‐diet interaction (Figure S4A,B, Supporting Information S2). In *App* ^NL‐G‐F^ mice, one of the top genes upregulated by PCS at 3 months in the cortex was *Nrgn*, encoding a calmodulin‐binding protein marker of synaptic degeneration in AD (Figure S5A) (Liu et al. 2020; Thorsell et al. 2010). Additionally, the stress‐related gene *Pou2f1*, encoding Oct‐1 (Stepchenko et al. 2021) (Figure S5B), was downregulated at 3 months, and mitochondrial ribosomal gene *Mrps35* was upregulated in PCS mice (Figure S5C). In the hippocampus at 12 months, PCS restored expression levels of *Cntn4* (Figure S5D), as well as increased expression of key APP cleavage‐regulating protein *Elavl4* and GABAergic marker *Gad1* (Figure S5E,F) (van der Linden et al. 2022).

We next performed GSEA on the transcriptome of PCS *App* ^NL‐G‐F^ mice to identify significantly altered pathways. In the cortex, we found that starting from 6 months, PCS *App* ^NL‐G‐F^ mice showed an increased expression of genes in pathways controlling synaptic processes and downregulation in inflammation (Figure 3D). In the hippocampus, we found that expression of genes in GABAergic synaptic function and ATP metabolism was increased while inflammatory processes were downregulated at 12 months (Figure 3E). In both brain regions, these changes counteracted the effects of *App* ^NL‐G‐F^ genotype seen in mice reared on the control diet.

As AD is a progressive disorder, we assessed whether PCS could alter the trajectory of gene expression. We used generalized linear models (GLM) to compare gene expression over time between WT and *App* ^NL‐G‐F^ mice stratified by perinatal diet. First, we fit a GLM using DESeq2, comparing the effect of genotype in control diet mice and identified genes with significantly changed expression (FDR < 0.05). We then determined the effect of genotype in PCS mice and identified genes that were not differentially expressed (FDR > 0.05). Intersecting these two lists, we found 2175 genes in the cortex and 3624 genes in the hippocampus that we dubbed choline‐protected genes, as the change in their expression due to the genotype of the *App* ^NL‐G‐F^ mice raised on the control diet was prevented by PCS (Figure 3F, Supporting Information S3).

One such choline‐protected gene in the cortex was neuron growth factor *Vgf* (Figure 3G). *Vgf* expression was downregulated in *App* ^NL‐G‐F^ mice (Three‐way ANOVA Genotype *F* (1,80) = 14.93, *p* = 0.0002), PCS dampened this downregulation, notably at 6 months (Three‐way ANOVA Genotype × Diet *F* (1,80) = 7.792, *p* = 0.0066). Another notably PCS‐protected gene was ribosomal gene *Rps23*, which was strongly upregulated in *App* ^NL‐G‐F^ mice (Three‐way ANOVA Genotype *F* (1,80) = 7.792, *p* < 0.0001). However, the rate of increase in its expression in PCS mice was significantly lower, suggesting that PCS protects against this upregulation (Figure 3H, Three‐way ANOVA Genotype × Diet *F* (1,80) = 4.128, *p* = 0.0445).

In the hippocampus, one notable choline‐protected gene was ubiquitin ligase *Ube3a*, whose downregulation is linked to synaptic dysfunction in AD (Olabarria et al. 2019). *Ube3a* was progressively downregulated in AD‐model mice (Figure 3I, Three‐way ANOVA Genotype *F* (1,80) = 9.106, *p* = 0.0034). PCS delayed the decline of *Ube3a* expression, and PCS mice had higher levels of its mRNA as compared to *App* ^NL‐G‐F^ mice raised on the control diet at all ages (Three‐way ANOVA Genotype × Diet *F* (1,80) = 5.369, *p* = 0.0231). One of the most prominent choline‐protected genes in the hippocampus was *H2‐DMa*, a member of the major histocompatibility complex II (MHC II) gene cluster. *H2‐DMa* was strongly upregulated in an age‐dependent fashion in *App* ^NL‐G‐F^ mice on the control diet (Figure 3J, Three‐way ANOVA Genotype *F* (1,80) = 33.27, *p* < 0.0001) and this effect was blunted by approximately half in PCS mice (Three‐way ANOVA Genotype × Diet *F* (1,80) = 5.834, *p* = 0.018). Interestingly, we found that when stratifying by sex, females showed stronger protection due to PCS than males (Figure S6A). One gene protected in females, but not males, in the hippocampus is a member of the *Ms4a* family, *Tmem176a* (Figure S6B). However, there are also genes with greater protection in males, such as *H2‐DMa*, which is choline‐protected in both sexes, but stronger in males (Figure S6C).

### 2.3 WGCNA Reveals Abnormal Aging and Novel Choline‐Responsive Clusters in *App* ^NL‐G‐F^ Mice

**FIGURE 4:**
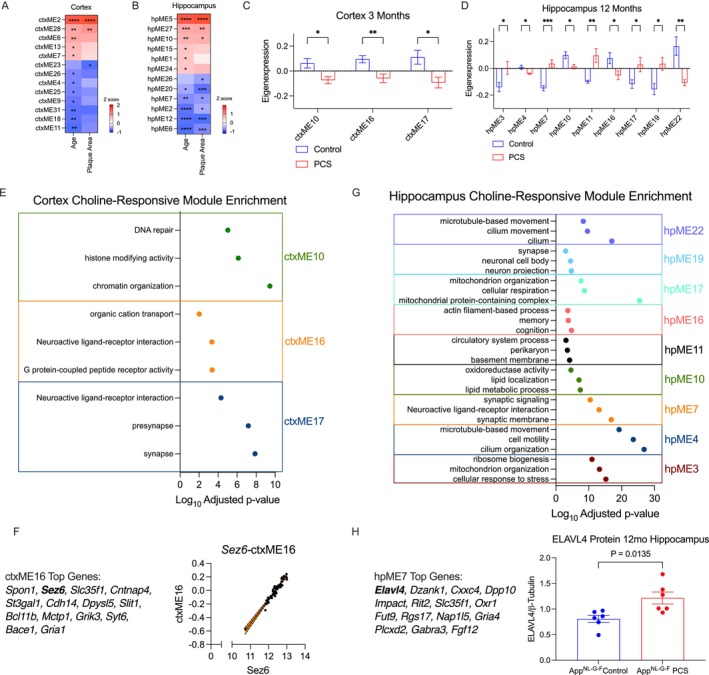
Weighted gene network correlation analysis reveals novel choline‐associated modules *App* ^NL‐G‐F^ mice. WGCNA was performed on *App* ^NL‐G‐F^ mice and module eigengenes (MEs) were correlated with age and plaque area in (A) the cortex and (B) hippocampus. Choline‐responsive modules were found by comparing eigenexpression of modules in control and PCS diet mice in (C) cortex and (D) hippocampus. (E) Choline responsive modules at 3 months in the cortex show downregulation of synaptic signaling and stress response, suggesting reduced hyperexcitability. (F) Correlation of ctxME16 gene *Sez6* with eigengene expression (G) Hippocampal choline‐responsive modules show upregulated synaptic function and stress response, while down regulating primary cilia function and lipid metabolism. (H) *Elavl4* is the top hub gene in choline‐responsive module hpME7 and is increased by PCS on the protein level. *p < 0.05, ** *p* < 0.01, *** *p* < 0.001, **** *p* 4.8 < 0.0001.

We next performed WGCNA to identify co‐expression module eigengenes (MEs) associated with disease progression and diet in *App* ^NL‐G‐F^ mice. In the cortex, we identified 35 co‐expression modules, while in the hippocampus, we identified 28 (Supporting Information S4). We first aimed to assess which MEs were associated with age and plaque accumulation during disease progression. In the cortex, 10 modules were associated with age alone (ctxME6, ctxME13, ctxME7, ctxME26, ctxME4, ctxME25, ctxME9, ctxME31, ctxME18, ctxME11), 1 was associated with plaque area alone (ctxME23), and 2 were associated with both plaque area and age (ctxME2, ctxME28) (Figure 4A). Shared age and plaque modules increase with age and were strongly enriched for glial immune responses (ctxME2) and interferon signaling (ctxME28), two hallmarks of AD (Figure S7A). The plaque‐associated ctxME23 was enriched for apoptosis and DNA binding. Modules positively associated with age alone were enriched for matrisome (ctxME13), remyelination (ctxME6), and translation (ctxME7). Meanwhile, modules negatively associated with age were enriched for ion channel function (ctxME11, ctxME9), synaptic structure (ctxME31), energy production (ctxME18), and proteostasis (ctxME26) (Figure S7A).

In the hippocampus, three modules were associated with age alone (hpME15, hpME1, hpME24), one was associated with plaque area alone (hpME26), and eight were associated with both traits (ME5, hpME27, hpME10, hpME20, hpME7, hpME2, hpME12, hpME6, Figure 4B). In the hippocampus, modules positively correlated with age, and plaques were enriched for inflammatory (hpME5), translation at synapse (hpME24), and lipid metabolism (hpME10) (Figure S7C). Meanwhile, downregulated modules were enriched for synapse organization/homeostasis pathways (hpME6, hpME7, hpME12, hpME2, hpME20). HpME26, which was negatively associated with plaques alone, was enriched for dendritic spine and glutamatergic synapse‐localized proteins. Lastly, modules positively correlated with age included pathways involved in interferon (hpME24), protein transport and AD (hpME1), and myelination (hpME15). Interestingly, when performing WGCNA on WT mice, we found fewer modules associated with age (Figure S7E,F), indicating that *App* ^NL‐G‐F^ mice have an abnormal aging phenotype.

To identify choline‐responsive modules, we compared eigengene expression values between control and PCS *App* ^NL‐G‐F^ mice at each age. In line with gene expression, the most choline‐responsive modules were found at 3 months in the cortex (ctxME10, ctxME16, ctxME17, Figure 4C, Figure S7B), and at 12 months in the hippocampus (hpME3, hpME4, hpME7, hpME10, hpME11, hpME16, hpME17, hpME19, hpME22, Figure 4D, Figure S7D). Interestingly, two hippocampal modules were altered by age, plaque area, and diet (hpME7 and hpME10), with PCS preventing age‐ and plaque‐dependent changes (Figure S7C).

Cortical choline‐responsive modules were enriched for synaptic function (ctxME16, ctxME17), chromatin remodeling, and DNA repair (ctxME10) (Figure 4E). Notably, synaptic and chromatin‐related pathways were downregulated in PCS mice, suggesting that PCS protects against early hyperexcitability found in AD models and prevents early epigenetic changes (Latif‐Hernandez et al. 2020). Specifically, ctxME16 contained seizure‐associated protein 6 (*Sez6*) as one of the top correlated genes, further suggesting reduced hyperexcitability (Spearman's rho = 0.93, *p* < 0.0001, Figure 4F).

In the hippocampus, PCS increased the expression of modules associated with cellular respiration (hpME3, hpME17), synaptic signaling (hpME7, hpME19), and basement membrane (hpME11) (Figure 4G). Conversely, PCS downregulated pathways for primary cilia function (hpME4, hpME22) and lipid metabolism (hpME10). Interestingly, one module containing the immediate early gene *Arc* was downregulated by PCS (hpME16). *Arc* is aberrantly overexpressed in AD and has been implicated in driving synaptic pathology (Kerrigan and Randall 2013). To further validate the effect of PCS, we investigated hpME7, the most significant choline‐responsive module (*p* < 0.001). The gene with the highest module membership was RNA binding protein *Elavl4*, which we previously identified as a top DEG changed by PCS at 12 months of age (Figure S5E). We assessed ELAVL4 protein levels by western blot and found a significant increase in PCS mice (*t* (10) = 2.995, *p* = 0.0135, Figure 4H), suggesting increased ELAVL4 coordinates upregulation of synaptic components in PCS mice.

### 2.4 Choline‐Protected Genes Identified in the Mouse AD Model Belong to a Set of Genes Associated With AD Diagnosis, Pathology, and Clinical Dementia Rating in Humans

**FIGURE 5:**
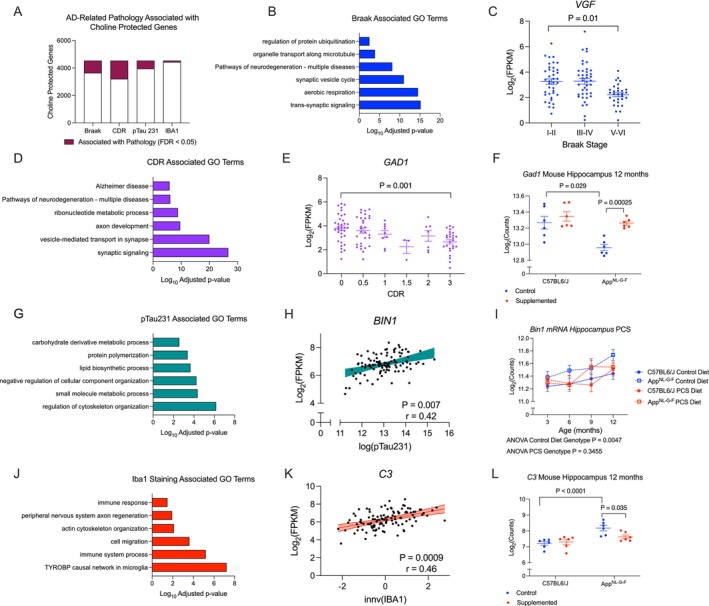
Choline protected genes are associated with measures of pathology and dementia in humans. (A) Number of choline protected genes associated with AD phenotypic measures in humans (FDR < 0.05). (B) Pathway enrichment of Braak associated choline‐protected genes reveals enrichment of synaptic signaling and aerobic respiration. (C) VGF is negatively associated with Braak stage. (D) Pathway enrichment of CDR‐associated choline protected genes reveals enrichment of synaptic signaling and Alzheimer's disease pathways. (E) *GAD1* expression decreases over increasing dementia severity in human AD. (F) PCS can prevent downregulation of *Gad1* at 12 months in *App* ^NL‐G‐F^ mouse hippocampus. (G) Pathway enrichment of pTau231‐associated choline‐protected genes reveals enrichment for cytoskeletal and metabolic processes. (H) AD risk gene *BIN1* is positively correlated with pTau231 levels in AD. (I) *Bin1* is significantly upregulated in *App* ^NL‐G‐F^ control diet hippocampus (ANOVA *p* = 0.0047), but is blunted in PCS mice (ANOVA *p* = 0.3455) (J) Pathway enrichment of IBA1‐associated choline‐protected genes reveals enrichment for TYROBP signaling, cytokine release, and cell–cell interactions. (K) Correlation of C3 with inverse normalized IBA1 staining. (L) PCS can dampen C3 upregulation at 12 months in the hippocampus of *App* ^NL‐G‐F^ mice.

Finally, we used the previously described transcriptomic data from autopsy samples of the dorsolateral prefrontal cortex of the participants of the Framingham Heart Study to investigate whether choline‐responsive genes found in the mouse model are abnormally expressed during human AD progression (Panitch et al. 2021, 2022). We found that of the 4530 human homologs of our choline‐protected genes, the expression of 886 was associated with Braak stage, 1328 with clinical dementia rating (CDR), 582 with pTau231, and 96 with IBA1 staining density, indicating that a high proportion of genes modulated by choline are associated with neuropathology and dementia in humans (Figure 5A, Supporting Information S5). Of the 888 genes associated with Braak stage, we found a strong enrichment for synaptic signaling and energy production (Figure 5B). The gene with the strongest effect size was *VGF*, which was also PCS‐protected (Figure 3G). Downregulated expression of *VGF* in AD was particularly evident in advanced disease (Braak I–II *n* = 39, III–IV *n* = 41, V–VI *n* = 30, Limma Adjusted *p*‐value = 0.01, Figure 5C).

With respect to genes associated with CDR, we observed a strong enrichment for synaptic function, oxidative phosphorylation, and AD‐related pathways, in line with the role of synaptic dysfunction in mediating cognitive decline (Figure 5D). One notable gene associated with CDR was *GAD1*, encoding the GABA‐synthesizing enzyme, glutamic acid decarboxylase, whose expression monotonically decreased over the CDR continuum, in line with deficits in inhibitory neurotransmission seen in AD (Figure 5E, *n* = 110, Limma logFC = −0.34, FDR = 0.001) (Xu et al. 2020). This deficit was also seen in *App* ^NL‐G‐F^ mice and was nearly completely reversed by PCS, suggesting normalized GABAergic signaling (Figure 5F, *n* = 6, PCS vs. Control in *App* ^NL‐G‐F^ mice, Wald Statistic = 5.12, FDR = 0.00025).

Genes significantly associated with pTau231 levels were mainly involved in microtubule dynamics, in line with the physiological function of tau (Figure 5G). AD risk factor *BIN1* was one of the top pTau231 associated genes (FDR = 0.007, Figure 5H). In the hippocampus of control diet mice, *Bin1* was consistently upregulated in *App* ^NL‐G‐F^ mice (Two‐way ANOVA Genotype *p* = 0.0047). However, in PCS mice, *Bin1* expression remained at wild type levels at 3, 6, and 12 months, ablating the effect of genotype (Two‐way ANOVA Genotype *p* = 0.3455, Figure 5I).

Genes significantly associated with IBA1 staining belonged mainly to the microglial TYROBP network, as well as migration, adhesion, and cytokine production (Figure 5J), including genes within the complement pathway, such as C3, which is upregulated in human AD and required for neurodegeneration in mouse models (Wu et al. 2019) (Figure 5K, *n* = 112, Pearsons *r* = 0.46, FDR = 0.0009). However, in mice, PCS partially dampened the upregulation of *C3* expression in the hippocampus of 12‐month‐old *App* ^NL‐G‐F^ mice (Figure 5L, *n* = 6, PCS vs. Control in *App* ^NL‐G‐F^ mice Wald Statistic = −3.38, FDR = 0.035).

## 3 Discussion

The data presented here show that the *App* ^*NL‐G‐F*^ AD model mice are characterized by age‐dependent transcriptomic abnormalities in the cerebral cortex and hippocampus involving mRNAs and lncRNAs, and that a large proportion of these changes is ameliorated by PCS. The abnormalities follow the time course of brain amyloid plaque accumulation, which triples between 3‐ and 9‐months of age and are consistent with previous reports showing enhanced inflammation, impaired synaptic function, and deficits in energy homeostasis in these mice (W.‐T. Chen et al. 2020; Daniels et al. 2023; Q. Li et al. 2024; Medina‐Vera et al. 2023; Naia et al. 2023). In addition to altered expression of protein‐coding genes, we identified several lncRNA species associated with AD progression. Two of the most prominent lncRNA transcripts shared between the cortex and hippocampus were *H2‐Q5* and *Ighd*, expressed from the MHC II and Igh loci, respectively. In *App* ^NL‐G‐F^ mice, the adaptive immune system is known to play a role in exacerbating AD pathology, and crossing AD model mice with *Rag2−/−* mice is reportedly associated with amelioration of cognitive deficits and neuropathology (Van Hoecke et al. 2024). In humans, the MHC locus produces several lncRNAs that fine‐tune the functions of immune cells (Chitnis et al. 2021; Gensterblum‐Miller et al. 2018). Moreover, microglia are major expressors of MHC II in the brain and potentially coordinate T‐cell responses in neurodegeneration (Perlmutter et al. 1992; Schetters et al. 2017). Additionally, lncRNA expression from the *IGH* locus regulates 3D chromosome conformation during VDJ recombination in B‐cells (Rothschild et al. 2020; Verma‐Gaur et al. 2012). While lncRNA species are typically not conserved between species, functional analogues may exist. We found in a human dataset that the AD‐associated lncRNA HCG22 controls similar genes to *H2‐Q5* based on a guilt‐by‐association approach, indicating that lncRNA expression from the MHC/H2 locus is modulated by and may coordinate microglia‐T‐cell interaction during AD pathology (Dai and Shen 2021). However, as the *App* ^NL‐G‐F^ model does not fully recapitulate AD pathology in humans, additional mechanistic validation of the role of these lncRNAs in AD is required.

The normalization of the brain gene expression patterns by PCS was robust, with approximately half of AD‐associated DEGs in the cortex and over 80% in the hippocampus of *App* ^NL‐G‐F^ animals reared on the control diet, losing statistical significance in PCS mice. While male and female *App* ^NL‐G‐F^ mice had similar transcriptomes, females tended to show a greater number of protected genes than males due to PCS, suggesting they may benefit more from this intervention. Mechanistically, as choline is a methyl donor, modulation of DNA and histone methylation could provide plausible mechanisms for how PCS can alter gene expression over the life of the animal. Indeed, in the accompanying paper, we provide evidence that the expression of many choline‐protected genes is correlated with altered DNA methylation (Krunic et al. 2025), suggesting that the delay in amyloid accumulation and amelioration of learning and memory deficits by PCS in these mice (Bellio et al. 2024) is mediated by this epigenetic process. This notion is supported by the observation that the largest number of differentially methylated CpG sites in DNA and differentially methylated genes associated with PCS in *App* ^NL‐G‐F^ mice was seen at 3 months of age, i.e., at the time most proximal to the treatment with supplemental dietary choline.

To determine the effects of the mouse genotype and PCS on brain gene expression patterns, we analyzed our data set with (1) DESeq2 to identify individual DEGs, (2) GSEA and WGCNA to identify biological pathways, and (3) intersecting genotype‐associated changes stratified by dietto identify choline‐protected genes that lose statistical significance in PCS mice. In the cortex, GSEA revealed that PCS upregulated synaptic signaling starting at 6 months and continuing towards 12 months. Moreover, we have shown that looking at choline‐protected genes reveals subtle changes in individual gene trajectory over time, reversing the effect of the AD model genotype. As these approaches use different criteria to identify changes, it suggests that more prominent changes in individual genes occur at 3 months in the cortex, while subthreshold (by FDR), but coordinated changes occur throughout later life, as shown by GSEA and choline‐protected genes. Likely, the lack of significance by FDR from 6 to 12 months is due to insufficient statistical power to detect subtle changes at an individual DEG level upon multiple testing correction. However, the use of more sensitive approaches reveals a robust resilience to AD‐like changes in PCS mice. One of the strongest changes observed at 3 months was *Nrgn*, which modulates synaptic strength and LTP, a process that is abnormal in the cortex of *App* ^NL‐G‐F^ mice already at this age (Latif‐Hernandez et al. 2020; Zhong et al. 2009). Previous studies showed that prenatal choline supplementation decreased the threshold for LTP in 3–4‐month‐old rats (Jones et al. 1999). As choline increases early synaptic strength and function, this may lead to a decreased propensity for synapses to deteriorate in response to AD pathology. PCS also prevented the decline in expression of cortical *Vgf*, encoding a precursor of peptides with protective actions in AD (Beckmann et al. 2020). Using WGCNA, we found that PCS reduces the expression of three MEs related to synapse‐enriched modules and epigenetic organization. In one module, ctxME16, genes highly correlated with the module include excitatory neurotransmission modulator *Sez6*, plaque component *Spon1* (Drummond et al. 2022), kainate receptors *Grik1* and *Grik3*, *Bace1*, as well as several synaptotagmin members such as *Syt6 and Syt10. Sez6* has been associated with AD and controls trafficking of kainate receptors (Pigoni et al. 2020). Interestingly, a link has been shown between kainate receptor activation on astrocytes and amyloidogenic processing, potentially providing a mechanism for reduced amyloidosis seen in PCS AD mice (Ourdev et al. 2019). Moreover, network hyperexcitability is a known early event in AD mice, and our data suggest that this downregulation in excitatory neurotransmission due to PCS may alleviate this phenotype (Kazim et al. 2017). Overall, our study suggests that PCS may increase synaptic strength while preventing network hyperexcitability in the cortex at early ages, leading to more stable synaptic function.

In the hippocampus, we found extensive transcriptomic changes at 12 months, demonstrating that early dietary intervention can protect against AD much later in life. One notable choline‐protected gene in the hippocampus was *H2‐DMa*, a member of the MHC complex. As previously discussed, *App* ^NL‐G‐F^ mice have increased expression of both mRNA and lncRNA species related to MHC II, and PCS may be able to prevent AD‐associated changes in antigen processing. Moreover, PCS prevents hippocampal downregulation of Angelman Syndrome gene, *Ube3a*. Loss of *Ube3a* is associated with decreased AMPA receptor activity, suggesting that prevention of *Ube3a* loss in PCS mice is related to preservation of excitatory signaling in AD (Greer et al. 2010). Using WGCNA, we further found that PCS can prevent changes in modules associated with synapse function, primary cilia, and lipid metabolism. The most highly upregulated module was hpME7, which was strongly enriched for synaptic function, with *Elavl4* as a major gene. Interestingly, *ELAVL4* was previously identified as a hub gene using WGCNA in human AD and its expression was decreased in the brains of symptomatic AD patients (Hu et al. 2020). *Elavl4* is an RNA‐binding protein that is upregulated in the hippocampus in response to learning and can stabilize synapses by modulating *Camk2a* mRNA (Quattrone et al. 2001; Sosanya et al. 2015). We further confirmed that ELAVL4 protein level was increased by PCS, suggesting that PCS can preserve hippocampal function through ELAVL4‐mediated stabilization of synaptic transcripts.

In our study, we noted that aside from 3 months in the cortex and 12 months in the hippocampus, we find very few DEGs reaching a 0.1 FDR cutoff when comparing PCS and control diets in *App* ^NL‐G‐F^ mice. Although our total cohort size included 96 mice, animals were assigned to 16 treatment groups (genotype, age, diet), leaving a modest 6 (3 males and 3 females) biological replicates per combination of independent variables. As the effect of perinatal choline is subtle compared to the effect of *App* ^NL‐G‐F^ genotype, our study may not be sufficiently powered to find individual PCS‐associated DEGs at each time point. Therefore, we used more sensitive downstream analysis methods, such as GSEA or by comparing the overall trajectory of gene expression over time based on diet, and found that PCS can subtly modulate many disease‐associated pathways in *App* ^NL‐G‐F^ mice. Thus, while individual gene expression effect sizes may not reach statistical significance, coordinated changes in pathway expression due to PCS can alter the overall phenotype of *App* ^NL‐G‐F^ mice towards resilience and resistance towards AD pathology.

Our present study has shown that many choline‐protected genes found in the AD mouse model are associated with core AD transcriptomic markers as well as measures of clinical impairment in AD patients, indicating that modulation of these genes by early‐life nutritional intervention may confer resilience and resistance to AD. A recent single‐cell transcriptomic study reported that choline metabolism may be upregulated in cognitively resilient individuals, as compared to cognitively impaired individuals, and there is considerable evidence that abnormal metabolism of choline‐containing phospholipids is a predominant metabolic defect in AD brain (Blusztajn and Slack 2023; Mathys et al. 2024). Moreover, choline intake is important in maintaining disease resilience throughout adulthood. In the Framingham Heart Study cohort, whose postmortem brain transcriptomic data set was used here, low choline intake was associated with increased risk of AD (Yuan et al. 2022). Additionally, circulating choline levels have been shown to associate with the progression of AD pathology in humans, highlighting the need to maintain choline levels throughout life (Judd et al. 2023). Likewise, a recent double‐blind, placebo‐controlled clinical trial has shown that choline supplementation in adults can reduce cognitive impairment in MC I (Jeon et al. 2024). Moreover, choline phospholipid levels are reduced in iPSC‐derived neurons harboring a loss‐of‐function AD risk gene, *ABCA7* (von Maydell et al. 2024). Our study corroborates the body of literature supporting choline as a potential accessible and low‐cost preventative measure against AD.

Overall, we have characterized brain transcriptomic changes across AD‐like disease progression in *App* ^NL‐G‐F^ mice on the level of mRNA and lncRNA and shown that early‐life choline supplementation induces resilience to these changes, suggesting that even the strong AD‐related endophenotypes that characterize this mouse model can be ameliorated by an early‐life nutritional strategy. In an accompanying paper, we provide evidence that PCS modulates brain DNA methylation patterns, acting as a donor of methyl groups, and propose that this epigenetic mechanism is responsible for the amelioration of AD‐related transcriptomic brain abnormalities in *App* ^NL‐G‐F^ mice. Our study further supports the notion that the formulation and implementation of public health policies designed to ensure adequate choline intake would be a valuable tool for the prevention of dementia and AD.

## 4 Methods

### 4.1 Ethics Statement

All procedures were conducted in accordance with the Animal Welfare Act (Animal Welfare Assurance Number A‐3316‐01) and to the principles of the National Institute of Health Guide for the Care and Use of Laboratory Animals (“The Guide”). All studies were approved by the Institutional Animal Care and Use Committee of Boston University.

### 4.2 Animals and Animal Care

C57BL/6J (WT) mice were purchased from Charles River Laboratories (Worcester, MA, USA) and then bred in‐house. Knock‐in *App* KM670/671NL (Swedish), E693G (Arctic), I716F (Iberian) (*App* ^NL‐G‐F^) mice were obtained from RIKEN BioResource Center under a Material Transfer Agreement and then bred in‐house. One week prior to mating, mice were placed on either a control AIN76A diet (#110098 Dyets Inc., Bethlehem, PA) consisting of carbohydrates (66.00%), protein (20.30%), and fat (5.00%)—provided as sucrose (500 g/kg), casein (200 g/kg), cornstarch (150 g/kg), cellulose (50 g/kg), corn oil (50 g/kg), mineral mix #200000 (35 g/kg), vitamin mix #300050 (10 g/kg), DL‐methionine (3 g/kg), and choline chloride (1.1 g/kg)—or on a choline‐supplemented AIN76A diet (#110184 Dyets Inc., Bethlehem, PA), differing only by containing 5.0 g/kg of choline chloride, creating control diet and choline‐supplemented groups. Homozygous *App* ^NL‐G‐F^ and C57BL/6J females were crossed correspondingly with homozygous *App* ^NL‐G‐F^ and C57BL/6J males. The pregnant dams continued to consume their specific prenatal diets through the birth of the litter and lactation until weaning of the pups on postnatal day 21 (Figure S1). Subsequently, all offspring consumed the control diet and water ad libitum and were randomly divided into four experimental age groups: 3, 6, 9, and 12 months of age. These ages were chosen as they represent when plaque pathology begins to accumulate in the cortex (3 months), the hippocampus (6 months), and plateaus (9–12 months). Animals were group housed by sex with a maximum of five females or four males per cage with controlled ambient temperature and humidity between 20°C–22°C and 50%–70%, respectively. Mice were housed on a reverse 12‐h light–dark cycle (11 p.m.–11 a.m. lights on). Mice were euthanized after completion of behavioral testing (Bellio et al. 2024) at 3, 6, 9, or 12 months of age (Figure S1).

### 4.3 Brain Processing and Brain Region Dissection

Mice were humanely euthanized 1 week after completion of behavioral testing at 3, 6, 9, or 12 months of age by CO_2_ inhalation followed by decapitation. Brains were quickly extracted and sectioned down the midline. The left hemisphere was immediately placed on ice in Leibovitz's L15 media (Gibco #41300‐039). Isolation of the entire hippocampus and a consistent cortical region encompassing motor, somatosensory, and cingulate cortices was completed using mouse surgical equipment and immediately frozen on dry ice and stored at −80°C until RNA extraction. The right hemisphere was immediately fixed in 10 volumes of PLP fixative (10 mM sodium periodate, 75 mM lysine, 4% paraformaldehyde; pH 7.4) for 24 h at 4°C, then cryoprotected in a graded series of 10% and 20% glycerol/2% dimethylsulfoxide in 0.1 M phosphate buffer, pH 7.3 solution for 24 h each.

### 4.4 RNA Extraction and RNA‐Sequencing

Frozen tissue from the hippocampus and cortex was used to extract total RNA and genomic DNA using the Zymo Quick RNA/DNA MiniPrep Plus Kit (Zymo D7003) under the manufacturer's conditions in batches of 32 samples that evenly represented all groups (age, sex, diet, genotype). Total RNA (1 μg) was sent to MedGenome Inc. (Foster City, CA, USA) for library preparation and sequencing. Library preparation was done using the Takara SMARTer Stranded Total RNA‐Seq Kit v2—Pico Input (Takara #634413). All samples passed quality control and were sequenced using 100‐nt paired‐end reads on an Illumina NovaSeq 6000 instrument with Illumina reagents to reach a minimum of 20 million paired‐end reads. All cortical samples were run in one lane, and all hippocampal samples were run in a separate lane to minimize batch effects.

### 4.5 Bioinformatic Analysis

Raw FASTQ files were used to trim adapter sequences and low‐quality bases (determined using a sliding window of 4 bases with an average below 20) using Trimmomatic (http://www.usadellab.org/cms/?page=trimmomatic). Quality and adapter‐trimmed FASTQ files were then aligned to the GRCm39 mouse transcriptome (https://ftp.ensembl.org/pub/release‐110/fasta/mus_musculus/cdna/) using SALMON (https://combine‐lab.github.io/salmon/about/). Quality control of the raw FASTQ files, trimmed FASTQ files, and aligned files was done using FASTQC (https://www.bioinformatics.babraham.ac.uk/projects/fastqc/). Differential gene expression analysis was completed using DESeq2 (Love et al. 2014). When doing differential gene expression analysis, covariates of sex, diet, age, and genotype were always included where appropriate.

### 4.6 Transcriptomic Comparison of Genotype

To understand the effects the *App* ^NL‐G‐F^ genotype has on gene expression across different ages, we conducted bulk RNA‐sequencing on both the cortex and hippocampus of wildtype and *App* ^NL‐G‐F^ mice ranging from 3‐ to 12‐month‐old at 3‐month intervals. We used 3 animals per group across each age, sex, genotype, and perinatal diet group, totaling 96 animals. We then compared WT mice to *App* ^NL‐G‐F^ mice using three genotype comparisons. First, we compared WT mice that received the control perinatal diet to *App* ^NL‐G‐F^ mice that also received the control diet perinatally. We then compared WT mice that received the choline‐supplemented diet perinatally to *App* ^NL‐G‐F^ mice that also received the choline‐supplemented diet perinatally. Lastly, we combined control and supplemented diet mice and compared wildtype and *App* ^NL‐G‐F^ mice, while controlling for diet in the model used to identify differentially expressed genes (DEGs). Additionally, these analyses were conducted while stratifying by sex. Each DEG needed to meet a false discovery rate (FDR) criterion of less than 0.1. We did this at each age while controlling for any sex effects, as well as in a combined model with all age groups that used age as a covariate in the model.

### 4.7 WGCNA

Weighted gene correlation network analysis (WGCNA) was conducted on hippocampal and cortical samples separately (Langfelder and Horvath 2008). Gene counts of all genes underwent a variance stabilizing transformation in DESeq2 to ensure homoscedasticity in the count matrix data. All genes were included in the analysis. The soft threshold for scale‐free network topology was determined by the lowest power value that achieved an *R* ^2^ value of 0.8. WGCNA was run with a signed network and topological overlap matrix (TOM) type to identify MEs. Pearson correlation coefficients were determined between MEs and sample traits, including age, perinatal diet across both genotypes, perinatal diet within *App* ^NL‐G‐F^ mice, and Aβ42‐positive percent area derived from immunohistochemical staining.

### 4.8 SDS‐PAGE and Western Blotting

Protein homogenates were generated from frozen hippocampal and cortical tissue using a sonicator and NP40 lysis buffer with Halt protease and phosphatase inhibitor cocktail (Thermo Scientific #78440). Protein concentrations were determined using a BCA assay. Protein was then diluted to a final concentration of 2.5 μg/μl in NP40 lysis buffer with 4× loading dye and beta‐mercaptoethanol, and 40 μg of protein was loaded into a 4%–12% polyacrylamide gel and run in MES buffer for 1 h and 20 min at 110 V. Proteins were transferred to a nitrocellulose membrane (Invitrogen #IB33001) using the iBlot 3 Western blot transfer device (Invitrogen #IB31001). For Iba1 immunoblotting, the membrane was blocked in 3% milk in TBS for 1 h at room temperature and then probed with a rabbit anti‐Iba1 antibody diluted in 3% milk in TBST overnight at 4°C (1:1000 Wako #016‐20001). The membrane was washed five times for 5 min each the next day and then incubated in a goat anti‐rabbit HRP conjugated antibody diluted in 3% milk in TBST for 1 h at room temperature (1:5000 Biorad #1706515), washed 5 times for 5 min each with TBST, incubated in SuperSignal West Pico Plus chemiluminescent substrate (Thermo Scientific #34580) for approximately 2 min, and imaged using a CCD camera. For GFAP and β‐actin, the above method was used except that the membrane was blocked in 5% milk in TBST, and the primary antibodies were diluted in 1% milk in TBST (mouse anti‐GFAP 1:1000, Cell Signaling Technologies #3670S; mouse anti‐beta‐actin 1:5000, Sigma‐Aldrich #A5316, ELAVL4 1:1000, Novus Biologicals H00001996‐M01), and the secondary antibody was diluted in TBST (goat anti‐mouse 1:2000, Biorad #1706516).

### 4.9 Human RNA‐Sequencing Data, AD Diagnosis, CDR, CERAD, and Braak Staging

Human transcriptomic data from the dorsolateral prefrontal cortex (Broadman area 8/9) from the Framingham Heart Study (FHS) were assessed to correlate choline‐responsive genes with AD diagnosis, clinical dementia rating, and AD pathologies. Briefly, total RNA was extracted and used for library preparation with a RIN > 5 cutoff before being sequenced on a next‐generation sequencing machine. Full details of library preparation, sequencing, quality control, mapping, and quantification of the human transcriptomic data are previously described (Panitch et al. 2021). Limma was used to associate RNA levels with AD pathophysiological traits, controlling for age, sex, and RIN (Ritchie et al. 2015). AD diagnosis was determined using the NIA‐Reagan criteria. Clinical dementia rating (CDR) was determined using previously described methods for FHS participants (Chouraki et al. 2015). Neuropathological assessment was performed using the procedures established by the Department of Veterans Affairs‐Boston University Brain Bank (Mez et al. 2015). CERAD scores were determined using the semi‐quantitative criteria for neuritic plaques previously published (Mirra et al. 1991). Braak staging was determined using standard neuropathological procedures (Braak et al. 2006). For analysis, a compressed Braak score was used where compressed Braak 1 was equal to Braak stages 0, 1, and 2; compressed Braak 2 was equal to Braak stages 3 and 4; and compressed Braak 3 was equal to Braak stages 5 and 6.

### 4.10 Human α‐Synuclein, Aβ40, pTau231 Protein Quantification

The same FHS cohort that was used for RNA‐sequencing was also used for ELISA protein quantification of α‐synuclein, Aβ40, and phosphorylated tau Thr231 (pTau231). α‐synuclein ELISA was conducted using previously described procedures (Adams et al. 2018). Aβ40 and pTau231 protein levels were measured using ELISA of homogenates from the dorsolateral prefrontal cortex gyral crest as previously described (Stathas et al. 2022). Inverse normal transformation was used for α‐synuclein and pTau231 measurements when correlating with gene expression.

### 4.11 Human Iba1 Staining

Iba1 staining of the dorsolateral prefrontal cortex was conducted as previously described (Friedberg et al. 2020). Inverse normal transformation was used for Iba1 density measurements when correlating with gene expression.

## Author Contributions

Concept and design: J.K.B., T.J.M. Acquisition of data: T.A.B., A.K., M.S.C., R.D., T.J.M., J.K.B. Data analyses: T.A.B., A.K., M.S.C., R.D., T.J.M., J.K.B., H.L., A.L. Interpretation of data: T.A.B., A.K., M.S.C., R.D., T.J.M., J.K.B., H.L., A.L., T.D.S. Drafting of the manuscript: T.A.B., A.K., J.K.B. Reviewing and editing: T.A.B., A.K., T.J.M., J.K.B., H.L., A.L., T.D.S.

## Conflicts of Interest

The authors declare no conflicts of interest.

## Supporting information


**Data S1.** acel70148‐sup‐0001‐Supinfo.zip.

**Data S2.** acel70148‐sup‐0002‐Supinfo.pdf.

## Data Availability

Code used to generate analysis can be found at https://github.com/krunican/RNAseq‐Code.
