# Supplementary material for: Perinatal Choline Supplementation Promotes Resilience Against Progression of Alzheimer's Disease‐Like Brain Transcriptomic Signatures in App NL‐G‐F Mice
Source: Aging Cell. 2025 Oct 13;24(11):e70148. doi: 10.1111/acel.70148 (PMC12608095; doi:10.1111/acel.70148)
Supplement: Supplementary file 2 — Data S2. acel70148‐sup‐0002‐Supinfo.pdf. [file ACEL-24-e70148-s002.pdf]

S1

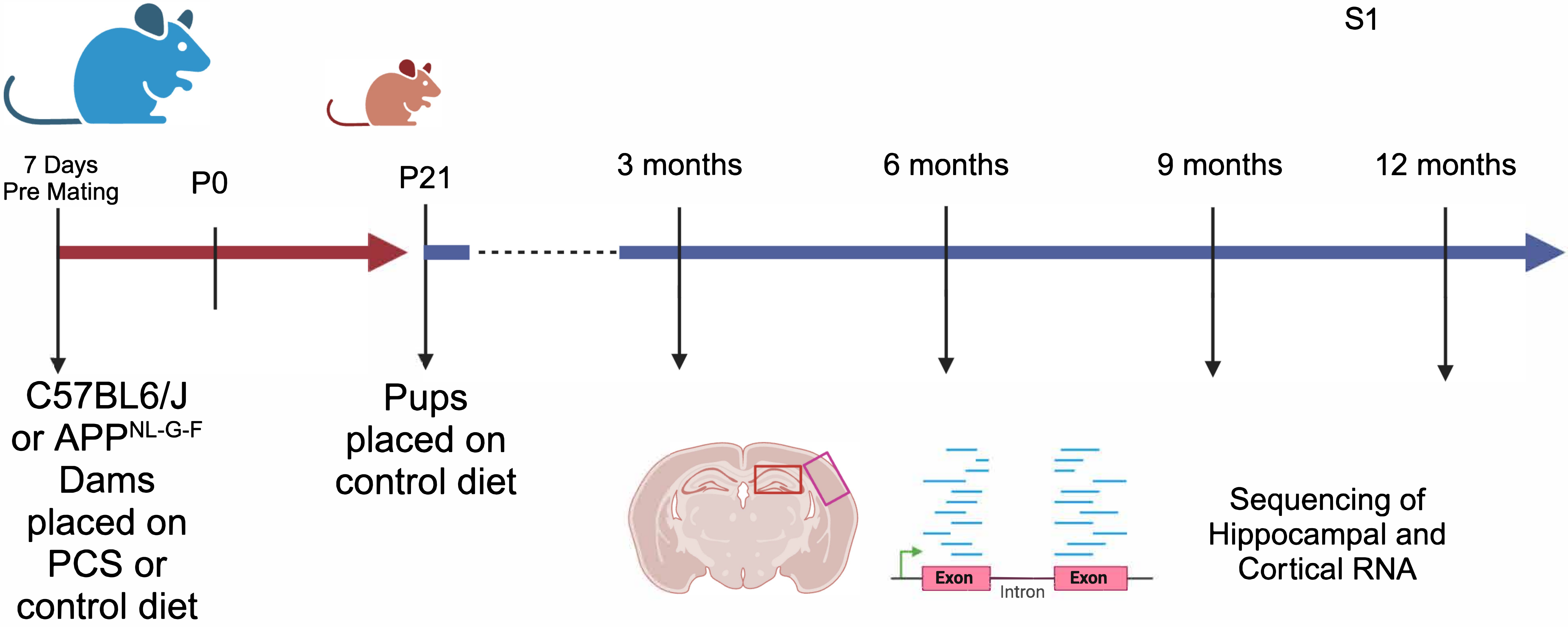

7 Days
Pre Mating
P0
P21
3 months
6 months
9 months
12 months
C57BL6/J or APP^{NL-G-F} Dams placed on PCS or control diet
Pups placed on control diet
Exon
Intron
Exon
Sequencing of Hippocampal and Cortical RNA

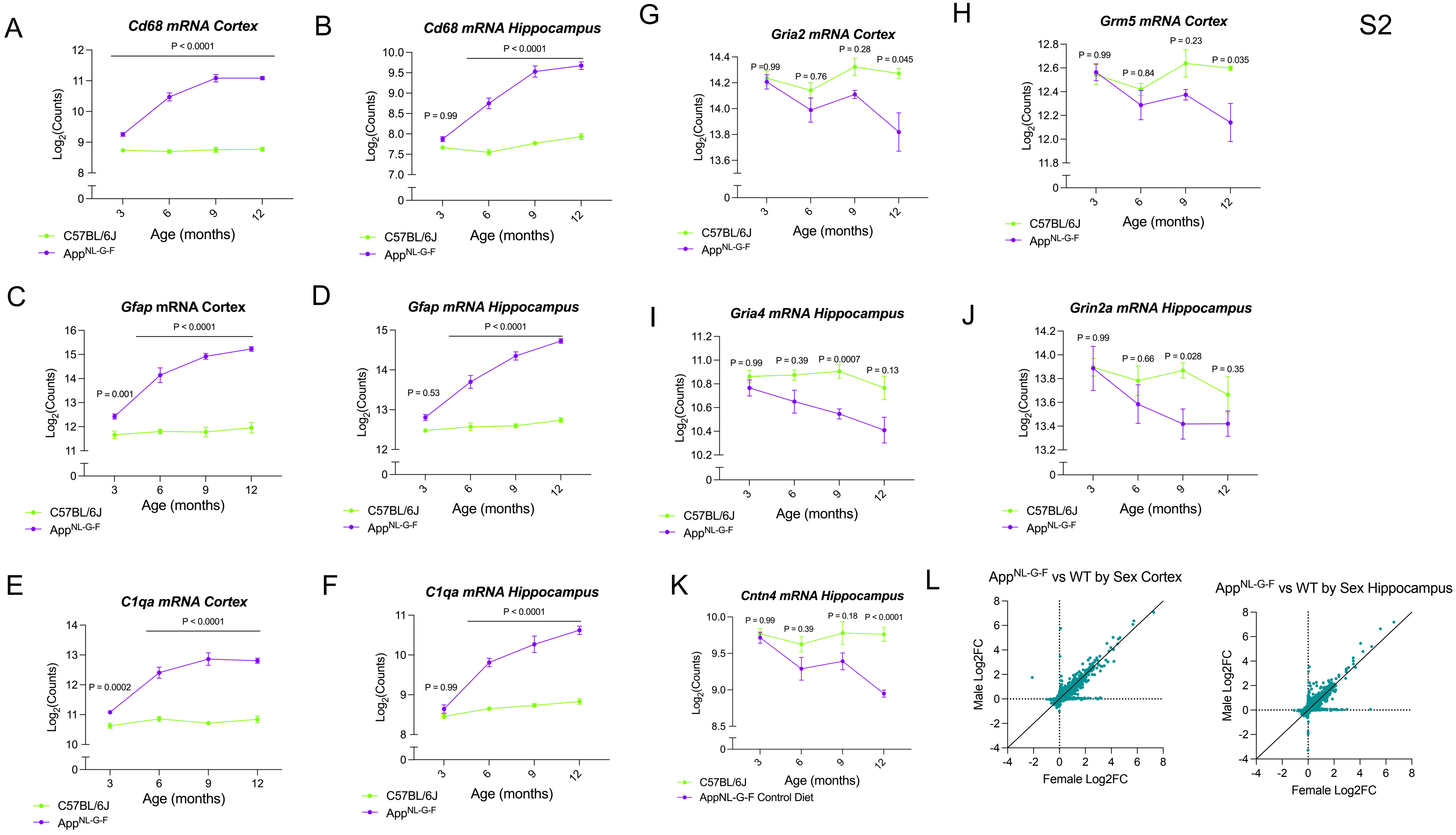
S2
A
Cd68 mRNA Cortex
P < 0.0001
Log2(Counts)
Age (months)
C57BL/6J
AppNL-G-F
B
Cd68 mRNA Hippocampus
P < 0.0001
P = 0.99
Log2(Counts)
Age (months)
C57BL/6J
AppNL-G-F
G
Gria2 mRNA Cortex
P =0.99
P = 0.76
P = 0.28
P = 0.045
Log2(Counts)
Age (months)
C57BL/6J
AppNL-G-F
H
Grm5 mRNA Cortex
P = 0.99
P = 0.84
P = 0.23
P = 0.035
Log2(Counts)
Age (months)
C57BL/6J
AppNL-G-F
C
Gfap mRNA Cortex
P < 0.0001
P = 0.001
Log2(Counts)
Age (months)
C57BL/6J
AppNL-G-F
D
Gfap mRNA Hippocampus
P < 0.0001
P = 0.53
Log2(Counts)
Age (months)
C57BL/6J
AppNL-G-F
I
Gria4 mRNA Hippocampus
P = 0.99
P = 0.39
P = 0.0007
P = 0.13
Log2(Counts)
Age (months)
C57BL/6J
AppNL-G-F
J
Grin2a mRNA Hippocampus
P = 0.99
P = 0.66
P = 0.028
P = 0.35
Log2(Counts)
Age (months)
C57BL/6J
AppNL-G-F
E
C1qa mRNA Cortex
P < 0.0001
P = 0.0002
Log2(Counts)
Age (months)
C57BL/6J
AppNL-G-F
F
C1qa mRNA Hippocampus
P < 0.0001
P = 0.99
Log2(Counts)
Age (months)
C57BL/6J
AppNL-G-F
K
Cntn4 mRNA Hippocampus
P = 0.99
P = 0.39
P = 0.18
P < 0.0001
Log2(Counts)
Age (months)
C57BL/6J
AppNL-G-F Control Diet
L
AppNL-G-F vs WT by Sex Cortex
Male Log2FC
Female Log2FC
AppNL-G-F vs WT by Sex Hippocampus
Male Log2FC
Female Log2FC

S3

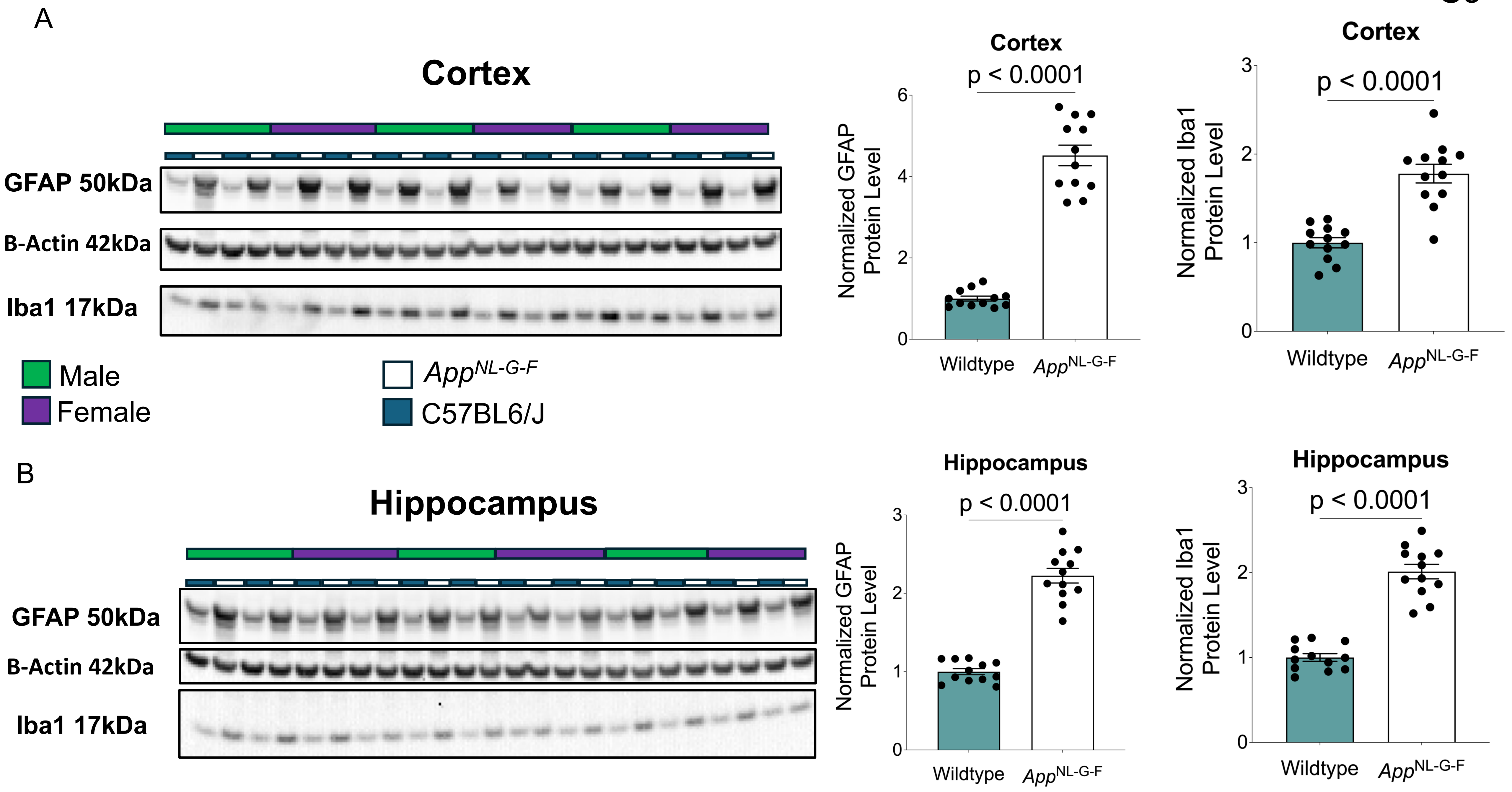

A
Cortex
GFAP 50kDa
B-Actin 42kDa
Iba1 17kDa
Male
Female
App^NL-G-F
C57BL6/J
Cortex
p < 0.0001
Normalized GFAP Protein Level
0
2
4
6
Wildtype
App^NL-G-F
Cortex
p < 0.0001
Normalized Iba1 Protein Level
0
1
2
3
Wildtype
App^NL-G-F
B
Hippocampus
GFAP 50kDa
B-Actin 42kDa
Iba1 17kDa
Hippocampus
p < 0.0001
Normalized GFAP Protein Level
0
1
2
3
Wildtype
App^NL-G-F
Hippocampus
p < 0.0001
Normalized Iba1 Protein Level
0
1
2
3
Wildtype
App^NL-G-F

S4

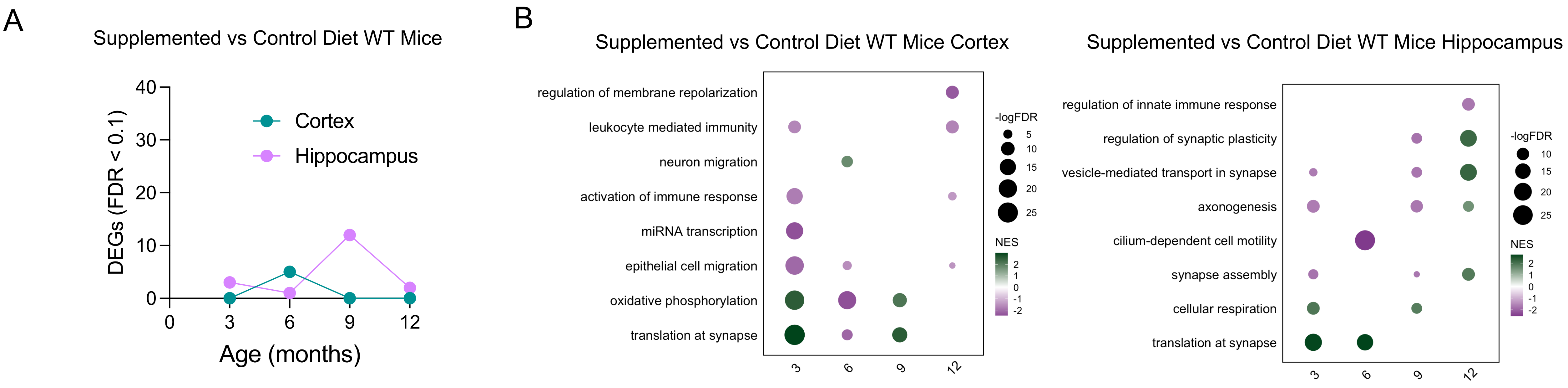
A
Supplemented vs Control Diet WT Mice
DEGs (FDR < 0.1)
Cortex
Hippocampus
Age (months)
B
Supplemented vs Control Diet WT Mice Cortex
regulation of membrane repolarization
leukocyte mediated immunity
neuron migration
activation of immune response
miRNA transcription
epithelial cell migration
oxidative phosphorylation
translation at synapse
-logFDR
NES
Supplemented vs Control Diet WT Mice Hippocampus
regulation of innate immune response
regulation of synaptic plasticity
vesicle-mediated transport in synapse
axonogenesis
cilium-dependent cell motility
synapse assembly
cellular respiration
translation at synapse

S5

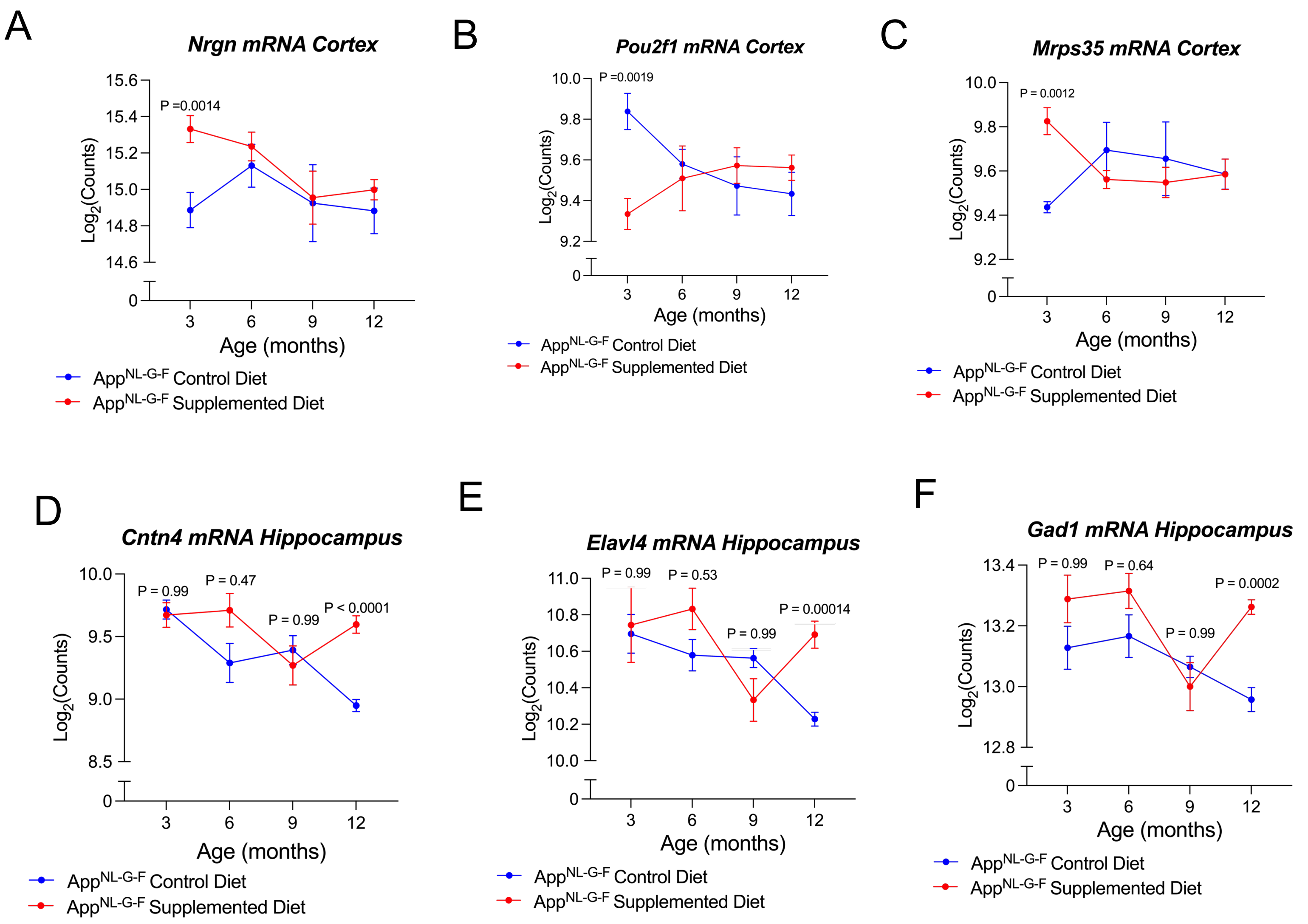

A
Nrgn mRNA Cortex
P =0.0014
Log2(Counts)
15.6
15.4
15.2
15.0
14.8
14.6
0
3
6
9
12
Age (months)
AppNL-G-F Control Diet
AppNL-G-F Supplemented Diet
B
Pou2f1 mRNA Cortex
P =0.0019
Log2(Counts)
10.0
9.8
9.6
9.4
9.2
0
3
6
9
12
Age (months)
AppNL-G-F Control Diet
AppNL-G-F Supplemented Diet
C
Mrps35 mRNA Cortex
P = 0.0012
Log2(Counts)
10.0
9.8
9.6
9.4
9.2
0
3
6
9
12
Age (months)
AppNL-G-F Control Diet
AppNL-G-F Supplemented Diet
D
Cntn4 mRNA Hippocampus
P = 0.99
P = 0.47
P = 0.99
P < 0.0001
Log2(Counts)
10.0
9.5
9.0
8.5
0
3
6
9
12
Age (months)
AppNL-G-F Control Diet
AppNL-G-F Supplemented Diet
E
Elavl4 mRNA Hippocampus
P = 0.99
P = 0.53
P = 0.99
P = 0.00014
Log2(Counts)
11.0
10.8
10.6
10.4
10.2
10.0
0
3
6
9
12
Age (months)
AppNL-G-F Control Diet
AppNL-G-F Supplemented Diet
F
Gad1 mRNA Hippocampus
P = 0.99
P = 0.64
P = 0.99
P = 0.0002
Log2(Counts)
13.4
13.2
13.0
12.8
0
3
6
9
12
Age (months)
AppNL-G-F Control Diet
AppNL-G-F Supplemented Diet

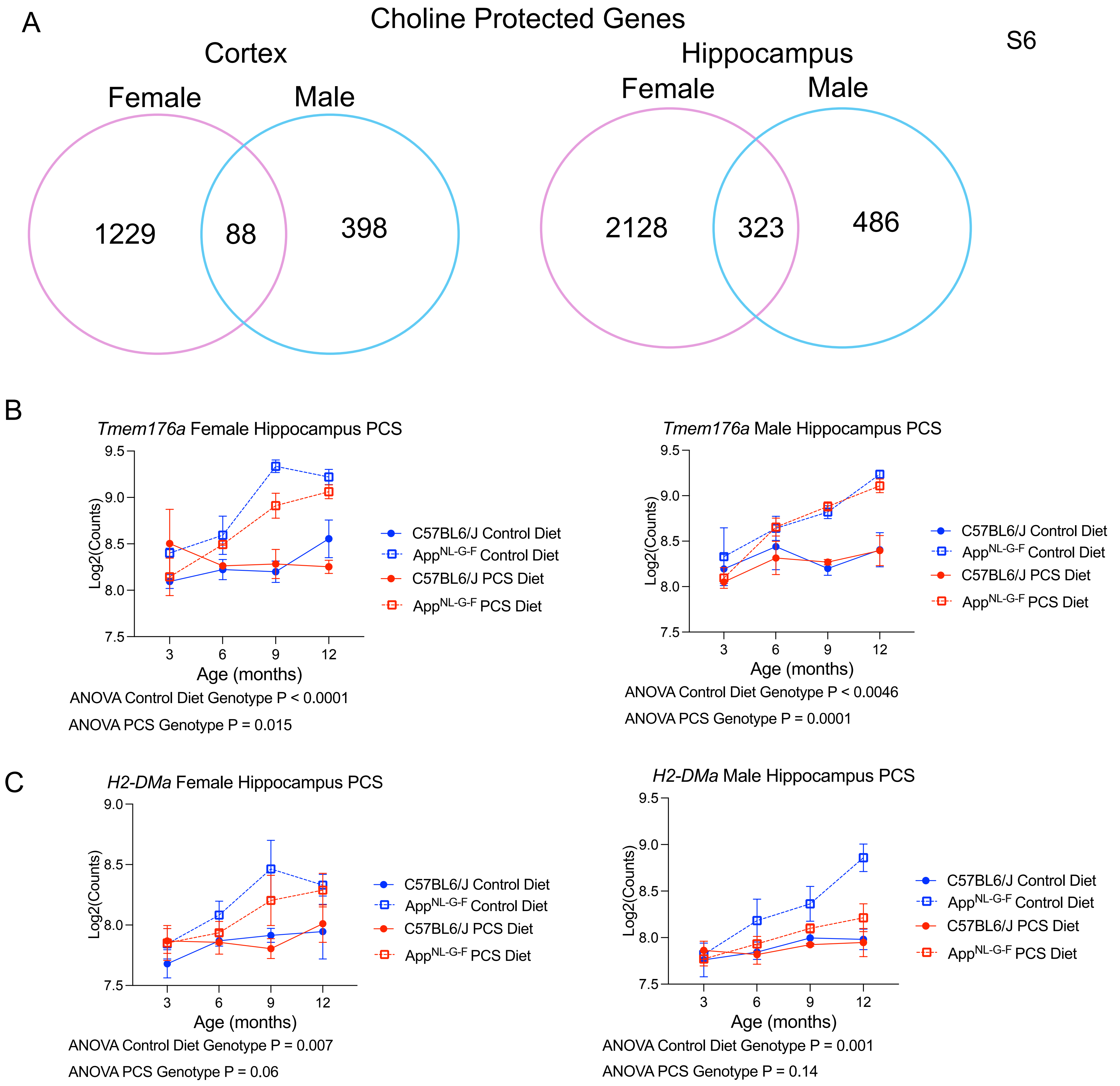
A
Choline Protected Genes
S6
Cortex
Female
Male
1229
88
398
Hippocampus
Female
Male
2128
323
486
B
Tmem176a Female Hippocampus PCS
Log2(Counts)
Age (months)
C57BL6/J Control Diet
AppNL-G-F Control Diet
C57BL6/J PCS Diet
AppNL-G-F PCS Diet
ANOVA Control Diet Genotype P < 0.0001
ANOVA PCS Genotype P = 0.015
Tmem176a Male Hippocampus PCS
ANOVA Control Diet Genotype P < 0.0046
ANOVA PCS Genotype P = 0.0001
C
H2-DMa Female Hippocampus PCS
ANOVA Control Diet Genotype P = 0.007
ANOVA PCS Genotype P = 0.06
H2-DMa Male Hippocampus PCS
ANOVA Control Diet Genotype P = 0.001
ANOVA PCS Genotype P = 0.14

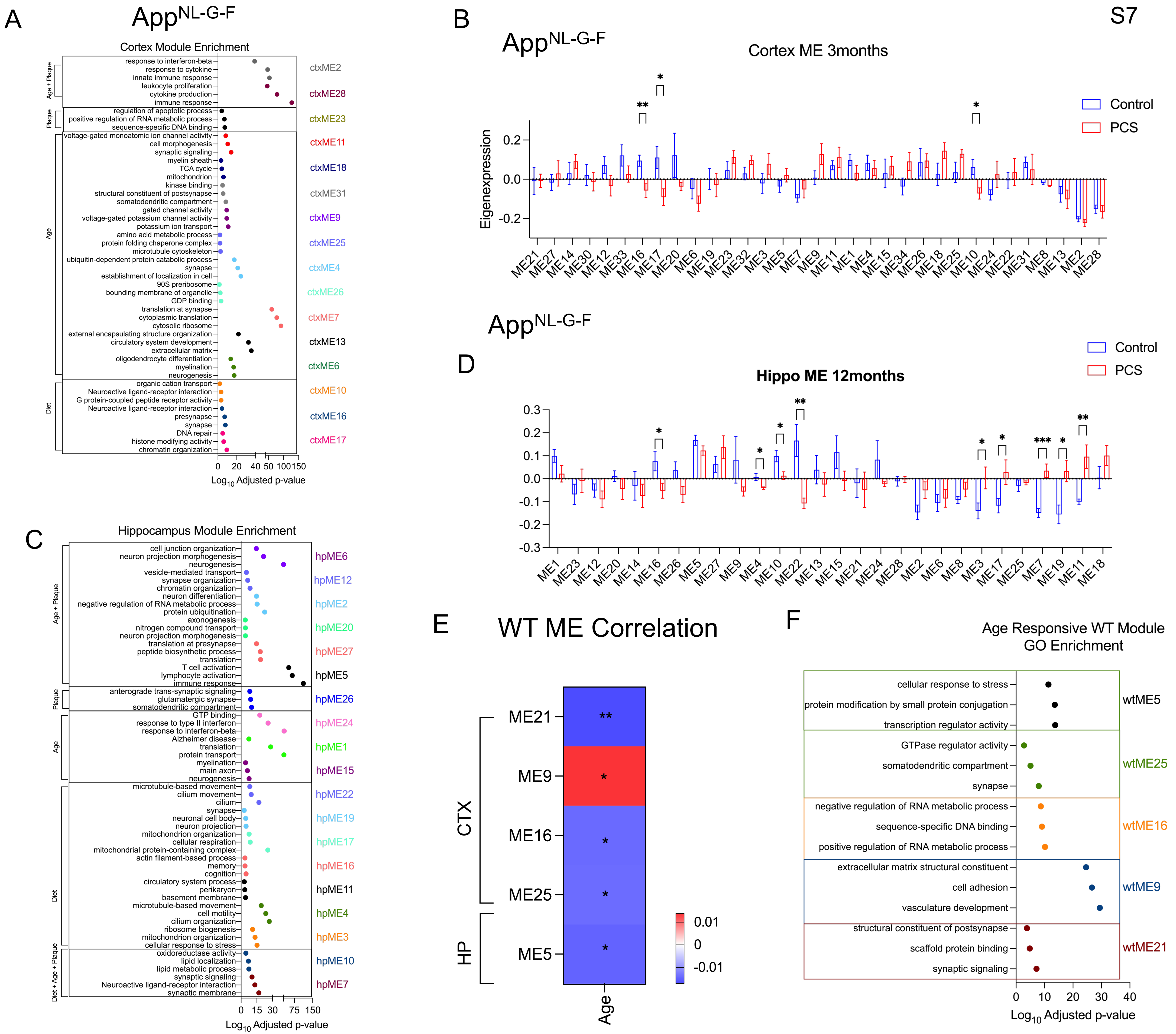
S7
A
App^NL-G-F
Cortex Module Enrichment
Age + Plaque
response to interferon-beta
response to cytokine
innate immune response
leukocyte proliferation
cytokine production
immune response
ctxME2
ctxME28
Plaque
regulation of apoptotic process
positive regulation of RNA metabolic process
sequence-specific DNA binding
ctxME23
Age
voltage-gated monoatomic ion channel activity
cell morphogenesis
synaptic signaling
ctxME11
myelin sheath
TCA cycle
mitochondrion
ctxME18
kinase binding
structural constituent of postsynapse
somatodendritic compartment
ctxME31
gated channel activity
voltage-gated potassium channel activity
potassium ion transport
ctxME9
amino acid metabolic process
protein folding chaperone complex
microtubule cytoskeleton
ctxME25
ubiquitin-dependent protein catabolic process
synapse
establishment of localization in cell
ctxME4
90S preribosome
bounding membrane of organelle
GDP binding
ctxME26
translation at synapse
cytoplasmic translation
cytosolic ribosome
ctxME7
external encapsulating structure organization
circulatory system development
extracellular matrix
ctxME13
oligodendrocyte differentiation
myelination
neurogenesis
ctxME6
Diet
organic cation transport
Neuroactive ligand-receptor interaction
G protein-coupled peptide receptor activity
ctxME10
Neuroactive ligand-receptor interaction
presynapse
synapse
ctxME16
DNA repair
histone modifying activity
chromatin organization
ctxME17
0 20 40 50 100 150
Log10 Adjusted p-value
B
App^NL-G-F
Cortex ME 3months
Control
PCS
Eigenexpression
0.2
0.0
-0.2
ME21 ME27 ME14 ME30 ME12 ME33 ME16 ME17 ME20 ME6 ME19 ME23 ME32 ME3 ME5 ME7 ME9 ME11 ME1 ME4 ME15 ME34 ME26 ME18 ME25 ME10 ME24 ME22 ME31 ME8 ME13 ME2 ME28
C
Hippocampus Module Enrichment
Age + Plaque
cell junction organization
neuron projection morphogenesis
neurogenesis
hpME6
vesicle-mediated transport
synapse organization
chromatin organization
hpME12
neuron differentiation
negative regulation of RNA metabolic process
protein ubiquitination
hpME2
axonogenesis
nitrogen compound transport
neuron projection morphogenesis
hpME20
translation at presynapse
peptide biosynthetic process
translation
hpME27
T cell activation
lymphocyte activation
immune response
hpME5
Plaque
anterograde trans-synaptic signaling
glutamatergic synapse
somatodendritic compartment
hpME26
Age
GTP binding
response to type II interferon
response to interferon-beta
hpME24
Alzheimer disease
translation
protein transport
hpME1
myelination
main axon
neurogenesis
hpME15
Diet
microtubule-based movement
cilium movement
cilium
hpME22
synapse
neuronal cell body
neuron projection
hpME19
mitochondrion organization
cellular respiration
mitochondrial protein-containing complex
hpME17
actin filament-based process
memory
cognition
hpME16
circulatory system process
perikaryon
basement membrane
hpME11
microtubule-based movement
cell motility
cilium organization
hpME4
ribosome biogenesis
mitochondrion organization
cellular response to stress
hpME3
Diet + Age + Plaque
oxidoreductase activity
lipid localization
lipid metabolic process
hpME10
synaptic signaling
Neuroactive ligand-receptor interaction
synaptic membrane
hpME7
0 15 30 75 150
Log10 Adjusted p-value
D
App^NL-G-F
Hippo ME 12months
Control
PCS
0.3
0.2
0.1
0.0
-0.1
-0.2
-0.3
ME1 ME23 ME12 ME20 ME14 ME16 ME26 ME5 ME27 ME9 ME4 ME10 ME22 ME13 ME15 ME21 ME24 ME28 ME2 ME6 ME8 ME3 ME17 ME25 ME7 ME19 ME11 ME18
E
WT ME Correlation
CTX
HP
ME21
ME9
ME16
ME25
ME5
Age
0.01
0
-0.01
F
Age Responsive WT Module GO Enrichment
cellular response to stress
protein modification by small protein conjugation
transcription regulator activity
wtME5
GTPase regulator activity
somatodendritic compartment
synapse
wtME25
negative regulation of RNA metabolic process
sequence-specific DNA binding
positive regulation of RNA metabolic process
wtME16
extracellular matrix structural constituent
cell adhesion
vasculature development
wtME9
structural constituent of postsynapse
scaffold protein binding
synaptic signaling
wtME21
0 10 20 30 40
Log10 Adjusted p-value

Supplemental Figure 1: Study Design 7 days prior to mating, female mice were placed on either a choline supplemented or standard diet and maintained until postnatal day 21, when pups were weaned and placed on the control diet. At 3-, 6-, 9-, and 12-months mice were sacrificed and RNA was extracted from the cortex and hippocampus.

Supplemental Figure 2: AppNL-G-F mice show increased inflammation and decreased synaptic function over time A-K) Normalized counts over time for main inflammatory and synaptic genes in AppNL-G-F and C57BL6/J mice (n = 6). FDR values calculated using DESeq2 Wald test. **L)** Comparison of log2 fold change between App^{NL-G-F} mice stratified by sex in the cortex and hippocampus, all genes shown. Data represented as mean +/- SEM

Supplemental Figure 3: Validation of increased GFAP and IBA1 in AppNL-G-F mice Western blot quantification of GFAP and IBA1 in AppNL-G-F and WT mice (n = 12). **A)** GFAP (Unpaired two-tailed t-test df = 22, t = 13.53, $p < 0.0001$) and IBA1 (Unpaired two-tailed t-test df = 22, t = 6.512, $p < 0.0001$) were significantly upregulated in the cortex of AppNL-G-F mice. **B)** GFAP (Unpaired two-tailed t-test df = 22, t = 12.07, $p < 0.0001$) and IBA1 (Unpaired two-tailed t-test df = 22, t = 10.49, $p < 0.0001$) were significantly upregulated in the hippocampus of AppNL-G-F mice.

Supplemental Figure 4: Gene expression changes in WT PCS mice
A) DEGs at each age (FDR < 0.1) in PCS versus control diet WT mice in the cortex and hippocampus. **B)** GSEA pathway enrichment of the whole transcriptome at each age in the cortex and hippocampus.

Supplemental Figure 5: PCS can prevent gene expression changes in AppNL-G-F mice A-F) Normalized counts over time for top DEGs in PCS AppNL-G-F mice (n = 6). FDR values calculated using DESeq2 Wald test. Data shown as mean +/- SEM

Supplemental Figure 6: Sex-specific effects of PCS in App^{NL-G-F} mice
A) Overlap between choline protected genes in males and females stratified by sex. **B)** *Tmem176a* as an example of a female-specific choline protected gene in the hippocampus. **C)** *H2-DMa* stratified by sex in males and females.

Supplemental Figure 7: WGCNA reveals coordinated changes in gene expression
WGCNA was performed on WT and AppNL-G-F mice separately. Pathway enrichment of modules associated with phenotypic features in the **A)** cortex and **B)** hippocampus in APP mice. **C)** Comparison of MEs at 3 months between control and PCS APP mice in the cortex. **D)** Comparison of MEs at 12 months between control and PCS APP mice in the hippocampus. **E)** Module trait correlation in WT animals reveals fewer age-associated modules than APP mice. **F)** Pathway enrichment of age-associated modules in WT mice.

File S1: Differentially expressed genes and GSEA pathway analysis for AppNL-G-F versus WT mice. Sheets stratified by age and brain region.

File S2: Differentially expressed genes and GSEA pathway analysis for PCS versus control diet comparisons. Sheets stratified by age and brain region.

File S3: List of choline-protected genes in the cortex and hippocampus. Log2 fold change and FDR in the AppNL-G-F versus WT group and AppNL-G-F versus control diet AppNL-G-F groups.

File S4: WGCNA eigengene module definitions, module membership correlations, and eigen-expression values stratified by brain region and genotype.

File S5: Human homologues of choline-protected genes and association with AD traits.
